# A Study of Nutritional Knowledge, Awareness, and Food Habits Among Indian Medical Professionals in the Context of the National Institute of Nutrition Guidelines, 2024

**DOI:** 10.7759/cureus.115122

**Published:** 2026-08-24

**Authors:** Rajesh Sharma, Vaishali Gautam, Himanshu Agrawal

**Affiliations:** 1 Community Medicine, GS Medical College and Hospital, Pilkhuwa, IND; 2 Pharmacology, GS Medical College and Hospital, Pilkhuwa, IND

**Keywords:** awareness and practice, continuing medical education, dietary preferences of doctors - nmc-cbme, nin dietary guidelines, nutritional knowledge

## Abstract

Background

Adequate nutritional knowledge and its translation into healthy dietary practice are essential prerequisites for medical professionals, who serve both as providers and as role models for the communities they serve. The National Institute of Nutrition (NIN) released comprehensively updated Dietary Guidelines for Indians in May 2024, integrating traditional Indian food knowledge with contemporary evidence and extended guidance to cooking methods and utensils. To our knowledge, no published study has assessed awareness of these revised recommendations across multiple strata of Indian medical professionals, nor has any study systematically compared trainees with settled professionals.

Methods

This analytical cross-sectional study was conducted among 222 Indian medical professionals at GS Medical College, Pilkhuwa, Uttar Pradesh, India, from May to November 2025, approximately one year after the release of the NIN-2024 recommendations. Participants were categorised as Trainees (MBBS students, interns, junior residents, and senior residents) and Settled (faculty, practising clinicians, and administrators). A validated, self-administered electronic questionnaire aligned with the NIN-2024 guidelines was used. Three composite indices: the Nutritional Knowledge Score (NKS, 22 items), the Guidelines Understanding Score (GUS, 9 items focusing specifically on NIN-2024 recommendations), and the Healthy Practice Score (HPS, nine behaviour-based items) were produced. Corresponding rates were computed as the percentage of the maximum possible score. Chi-square tests, independent t-tests, one-way ANOVA, and multiple linear regression were applied using IBM SPSS Statistics for Windows, Version 26 (Released 2018; IBM Corp., Armonk, NY, USA).

Results

The mean NKS was 10.82 ± 3.3, yielding an overall nutritional knowledge rate of 49.18%; the majority of participants (55%) demonstrated only average nutritional knowledge, and merely 2.7% achieved a good NKS. In contrast, the guidelines understanding rate was considerably higher at 71.22% (mean GUS 6.41 ± 2.2), indicating greater familiarity with recent updates than with holistic nutritional concepts. The overall healthy practice rate was 53.2% (mean HPS 4.79 ± 1.7). Faculty members recorded significantly higher NKS than all other strata; women, participants in their 30s and 40s, and those consuming home-prepared meals consistently outperformed their counterparts across knowledge and practice domains. In multiple linear regression, the source of food emerged as a significant independent predictor of HPS (β = -1.231, p < 0.001), while settlement status did not independently predict HPS (β = -0.071, p = 0.838) after accounting for dietary source.

Conclusions

Nutritional knowledge among Indian medical professionals remains insufficient and is inconsistently translated into practice, irrespective of training status. The comparatively stronger awareness of NIN-2024 updates points to a systemic imbalance in nutritional education - one that emphasises guideline dissemination over foundational competency. Healthy dietary behaviour was determined more by the source of food than by professional seniority or settlement status. Addressing these lacunae necessitates a multipronged approach: curriculum reform aligned with the NMC-CBME framework, structured nutrition training across all career stages, and policy-level attention to the nutritional quality of institutional food environments.

## Introduction

Adequate nutrition and good dietary habits are paramount for sustaining good health. These are influenced by an individual’s environment, resource availability, customs, and knowledge of nutritional facts [1-3]. Recent changes in work culture and the rise of consumerism have changed our dietary habits. The easy availability of High-Salt, High-Sugar (HSHS) Ultra-Processed Food (UPF) has altered our dietary preferences through complex, non-univocal mechanisms [4,5]. Recognising these changes, the National Institute of Nutrition (NIN) released recommendations in 2024 in a publication entitled "Dietary Guidelines for Indians" [6]. These guidelines move beyond prescriptive lists and now reflect extensive research that integrates traditional Indian food knowledge with modern science [6]. They now focus on improving vegetable intake, decreasing sugar consumption, and placing greater emphasis on whole food consumption. These new guidelines reflect efforts to address the dual burden of undernutrition and the rapid rise of lifestyle diseases in India, and now even give directions on cooking methods and utensils [6,7].

Understanding these dietary guidelines is of profound importance for Indian medical professionals, not only because they serve as the fountainhead of knowledge for the communities they serve but also because they function as role models within those communities. Awareness of nutritional guidelines has been extensively studied in varied population groups in India, such as pregnant females [8], people with chronic diseases [9,10], adolescents [11,12], and Indian adults [13]. However, only a few studies [14-16] have explored the understanding of nutritional guidelines among healthcare workers, and none of these studies has incorporated the recent revisions outlined by the NIN in 2024. Moreover, existing literature is further limited by inadequate representation, as prior studies have focused exclusively on either young medical students or practising professionals in isolation, thereby lacking the capacity to capture a holistic understanding of the strengths and weaknesses inherent across these distinct professional groups.

Thus, it is imperative to assess the current understanding of dietary guidelines among Indian medical professionals in relation to their dietary choices. We, therefore, aim to evaluate the nutritional knowledge, including current updates and nutrition-related practices, among Indian medical professionals associated with GS Medical College, Pilkhuwa, India.

## Materials and methods

Study design and setting

We conducted an analytical cross-sectional study among Indian medical professionals at GS Medical College. GS Medical College and Hospital is a private medical college in Pilkhuwa, Uttar Pradesh, India. It has pan-India representation within the medical fraternity, with both students and working professionals coming from various states of India. Undergraduate students are required to stay in a hostel, which is not the case for working medical professionals. We recruited the participants from May 2025 to November 2025, approximately one year after the release of the NIN-2024 recommendations, and used a self-administered questionnaire delivered electronically via a Google Forms link (Google, Inc., Mountain View, CA, USA).

Study participants

For this study, Indian medical professionals comprised undergraduate medical students, residents (junior residents, postgraduate students, and senior residents), faculty (medical academicians), and practising clinicians associated with GS Medical College. We categorised the study population into two groups: Trainees and Settled. Trainees included those who were pursuing medical education (MBBS students and interns), junior residents (those who had completed their medical degree but had not yet completed postgraduate training), and senior residents (those who had completed both undergraduate and postgraduate training). This represented the younger group who were pursuing the medical curriculum or had just started specialisation and hence whose work was largely supervised. The Settled group included faculty members (academicians), practising clinicians, and medical professionals currently involved in administrative roles in the study area, i.e., GS Medical College and Hospital. All students and medical professionals from all strata of professional roles at GS Medical College and Hospital were eligible to participate. Non-consenting individuals, those aged under 18 years, and those who were unavailable during the study period due to medical reasons or travel were excluded.

Study tool and data collection

The questionnaire was designed in line with the NIN 2024 recommendations (Appendix 1) [6]. All questions were appropriately modified based on a prior validation survey conducted with 20 Indian medical professionals. The Google Form link was shared during academic gatherings and through social media groups. Completion was voluntary. Although sampling was convenience-based, medical professionals working in all departments and health facilities, as well as residents and students belonging to all admission years, were approached to enhance representativeness.

**Figure 1:**
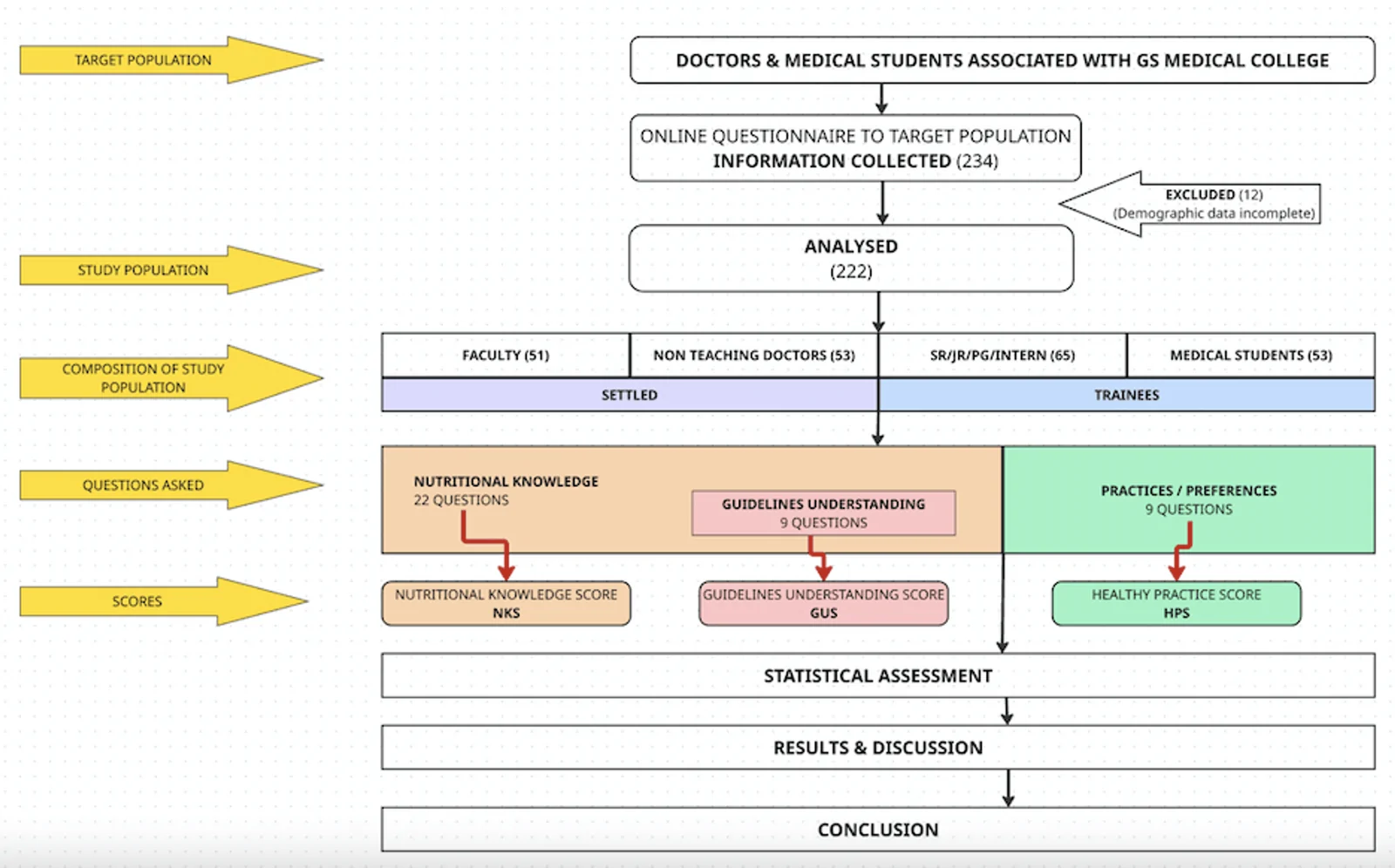
Detailed methodology for data collection and analysis NKS: Nutritional Knowledge Score; GUS: Guidelines Understanding Score; HPS: Healthy Practice Score

Data were collected, and scoring was subsequently performed. Scoring was conducted under three domains: ‘Nutritional Knowledge Score’ (NKS), ‘Guidelines Understanding Score’ (GUS), and ‘Healthy Practice Score’ (HPS), as explained in Figure 1. 

Nutritional knowledge was assessed using the NKS, derived from one point awarded to each correct response to 22 questions and graded as Good (>75%), Average (50%-75%), or Poor (<50%). Of these 22 questions, nine questions focusing on the new updates in NIN 2024 recommendations were similarly used to calculate the ‘GUS’. The 'HPS' was computed from responses to nine questions assessing behaviour consistent with the NIN 2024 recommendations, with one point awarded for each healthy practice and zero for unhealthy practices. As the study was conducted in a residential college, we examined whether sourcing food from one’s own kitchen influenced NKS or HPS compared with consuming food from the hostel mess.

We also estimated the ‘Rate’, which was calculated as the percentage of correct responses for a given score, by dividing the mean score obtained by the maximum possible score, as stated by Scalvedi et al. [17]. A rate of 100 indicated that all questions related to that index were answered correctly.

Sample size

Nutritional knowledge formed the basis for calculating the sample size. The sample size was estimated assuming that 50% of participants would have good nutritional knowledge. At a 95% confidence level and with an absolute precision of 7%, the required sample size was calculated to be 196 using the formula $n=z^{2}pq/d^{2}$. Allowing for a design effect of 1.1, the final target sample size was set at a minimum of 216 respondents.

We used IBM SPSS Statistics for Windows, Version 26 (Released 2018; IBM Corp., Armonk, NY, USA) for analysis. Chi-square tests were applied to compare proportions. Mean differences for continuous variables were compared using independent two-sample t tests. One-way ANOVA was employed to test the significance of the variance across independent means. The primary outcome variable was the “rate” of NKS, GUS, and HPS.

The study was conducted according to the ICMR Ethical Guidelines for Research on Human Participants (2017) [18]. All procedures involving research study participants were approved by the Ethical Committee of GS Medical College vide Certificate No. GSMCH/2025/IEC/01. The target population was informed about the nature and purpose of the study, and assurance of anonymity was provided in the online form.

## Results

A total of 234 responses were obtained. Twelve responses were excluded as they lacked a full demographic profile, and the remainder were analysed, as already explained in Figure 1. The mean age of respondents was 37.2 ± 15.2 years. Gender distribution was comparable. Of the total participants, 118 (54.2%) were trainees. The primary source of food was the home kitchen for 120 respondents (54.1%), while the remainder consumed food from the hostel mess. Detailed sociodemographic details are depicted in Table 1.

**Table 1:** Sociodemographic characteristics of respondents (n=222) LD: Lifestyle Diseases; SR: Senior Resident; JR: Junior Resident; PG: Postgraduate

Sociodemographic parameter	Categories		N (Count)	% (percentage)
Sex	Male		105	47.3
	Female		117	52.7
Age groups	20-26		54	24.3
	26-31		58	26.1
	31-49		55	24.8
	49-67		55	24.8
Current responsibility	Settled	Faculty	51	23.0
		Non-teaching doctors	53	23.9
	Trainees	SR/JR/PG/Intern	65	29.3
		Medical student	53	23.9
Regular source of food	Home Kitchen		120	54.1
	Hostel Mess		102	45.9
Lifestyle disease burden	Moderate - High (2 or more have LSD) - LD High		112	50.5
	Nil - Low (0-1 member has LSD) - LD Low		110	49.5

Mean nutritional score was 10.8 ± 3.3; however, only 36.5% knew that cut vegetables should not be washed thoroughly before cooking, and only 15.3% correctly identified that sunflower oil is not a good source of n-3 PUFA (polyunsaturated fatty acid). With respect to Guideline-Specific Awareness, the mean score was 6.4 ± 2.2; however, only 15.8% knew that both diet and physical activity can reduce diabetes risk by 80%, and only 28.4% knew the recommended vegetable intake (400 g/day). A mean score of 4.8 ± 1.7 was observed for the Healthy Preference and Practice Score. Only 6.8% consumed sprouted beans more than three times a week, and only 16.2% practised yoga more than three times weekly. Brisk walking/exercise for >45 min three times weekly was achieved by only 42.3%. Detailed observations from participants are provided in Table 2.

**Table 2:** Questionnaire questions, expected responses, and responses Guidelines awareness (B) = 6.41 ± 2.2; Nutritional knowledge (A+B) = 10.82 ± 3.3; Preference/practice = 4.79 ± 1.7. PUFA: polyunsaturated fatty acid; MUFA: monounsaturated fatty acid; SFA: saturated fatty acid; TFA: trans fatty acid

Domain	Questions (22)	Expected response/practice	% Correct
A) Nutritional knowledge	Nutri cereals and legumes are good sources of essential fatty acids	Correct	70.7
	Refined oils have better nutritional value than non-refined oils	Incorrect	65.3
	Nonstick (Teflon-coated) cookware is good as it can withstand high temperatures	Incorrect	68.9
	Oils containing n-6 PUFA (like safflower) are good and can be taken in plenty	Incorrect	49.5
	Cut vegetables should be thoroughly washed before cooking	Incorrect	36.5
	Fortifying ultra-processed food can make it wholesome and healthy	Incorrect	63.5
	Spices are essential for good health	Correct	55.5
	Sunflower oil is not a good source of n-3 PUFA	Correct	15.3
	The size of the sprout for the best nutritional value should be equal to the kernel	Correct	29.7
	Tea/coffee can be taken in moderation	Correct	31.1
	Tea/coffee should be avoided for one hour after a meal	Correct	36.5
	Dietary fibre is nutritionally inactive	Incorrect	55.0
	Microgreens are good sources of amino acids & fatty acids	Correct	55.9
B) Recommendations emphasised in NIN 2024 guidelines	Table sugar is an essential dietary component	Incorrect	73.4
	Mixed fruit juices have been recommended	Incorrect	67.6
	Ready-to-eat bakery products are a good source of nutrition and are recommended	Incorrect	91.0
	Baking soda can be added to pulses to facilitate proper cooking	Incorrect	67.6
	In the food pyramid, fruits & veg. form the base	Correct	42.8
	A healthy adult is recommended to consume 400 gm of vegetables per day	Correct	28.4
	A healthy diet and physical activity can reduce diabetes by 80%	Correct	15.8
	Earthenware cookware has been considered the best	Correct	35.1
	Indians consume fewer vegetables than recommended	Correct	18.9
Preference/practice questions	Preference for fruits over fruit juices	Fruits	83.3
	Frequency of consumption of bakery products/Halwai-made products	< Once a week	52.3
	Frequency of brisk walk/moderate exercise lasting > 45 minutes in a week	> Thrice a week	42.3
	Frequency of Yoga in a week	> Thrice a week	16.2
	Frequency of consumption of Tea/coffee in a day	2-4 cups/day	73.4
	Frequency of consumption of sprouted beans in a week	> Thrice a week	6.8
	Frequency of ordering food from outside in a week	< Once a week	57.2
	Going out for eating less than once a week	< Once a week	70.3
	Cooking medium used in the kitchen	Containing PUFA + UFA, No SFA/TFA	77.5

NKS ranged from 0 to 18. The overall nutritional knowledge rate was 49.2%. The majority (55%) demonstrated average NKS. Only six respondents (2.7%) who answered more than three-fourths had good NKS. Significantly higher mean NKS values were observed among women, participants in their 30s and 40s, and faculty members (11.6, 11.9, and 12.2, respectively). Respondents who consumed food primarily from the home kitchen also demonstrated significantly higher mean NKS (11.3). The respondents’ GUS ranged from 0 to 8, and the mean ± SD was 6.41 ± 2.2. The overall guidelines understanding rate was 71.2%. Significantly higher mean scores were recorded among women, faculty members, and participants in their 30s and 40s (6.97, 7.31, and 7.13, respectively). With regard to practice, the respondents’ HPS ranged from 0 to 9, with a mean of 4.79 ± 1.7. The overall healthy practice rate was 53.2%. Significantly higher scores were observed among participants in the Settled group and those who consumed food primarily from the home kitchen (Table 3). Calculation of the rates of NKS, GUS, and HPS is shown in Table 4.

**Table 3:** Variation of mean NKS, NIN guidelines awareness and practice scores with sociodemographic variables All values were obtained using the one-way ANOVA test. *p < 0.05 indicates statistical significance; ^a^F-value from the one-way ANOVA test. NKS: Nutritional Knowledge Score; GUS: Guidelines Understanding Score; HPS: Healthy Practice Score; ANOVA: Analysis of Variance

Variables	Categories	NKS mean value	F-value^a^	p-value	GUS mean value	F-value	p-value	HPS mean value	F-value	p-value
Gender	Male	9.9	14.3	0.0002*	5.8	17.0	0.0001*	4.7	0.62	0.4419
	Female	11.6			6.9			4.9		
Age Group	20-26	10.4	2.9	0.032*	6.3	2.8	0.043*	4.5	11.7	0.000*
	26-31	10.5			6.1			4.0		
	31-49	11.9			7.1			4.9		
	49-67	10.4			6.1			5.8		
Current Responsibility	UG Students	10.5	4.1	0.007*	6.4	4.3	0.005*	4.7	10.2	0.000*
	Interns/PG/SR	10.5			6.2			3.9		
	Faculty	12.2			7.3			5.0		
	Practitioners/Administrator	10.1			5.9			5.6		
Food Source	Home Kitchen	11.3	5.4	0.023*	6.7	3.4	0.067	5.4	34.8	< 0.0001*
	Hostel Mess	10.3			6.1			4.1		

**Table 4:** Calculation of rate of nutritional knowledge, NIN guidelines awareness and practice scores Rate is calculated according to Scalvedi et al. [17].

Descriptives	Nutritional Knowledge Score (NKS)	Guidelines Understanding Score (GUS)	Healthy Practice Score (HPS)
Min in the sample	0	0	0
Max in the sample	18	8	9
SD	3.3	2.2	1.7
Theoretical Max (b)	22	9	9
Mean (a)	10.8	6.4	4.8
Rate (a)/(b) %	49.2	71.2	53.2

Table 5 compares nutritional knowledge categories across respondent responsibilities. A significant difference was observed between the faculty group and other categories. Of the six respondents with good NKS, five belonged to the faculty group. This group also had the lowest proportion of respondents with poor NKS (27%).

**Table 5:** Nutritional Knowledge Score (NKS) of respondents All values obtained using the Chi-square test. SR: Senior Resident; JR: Junior Resident; PG: Postgraduate

Responsibilities of respondents	Grade of NKS score			Chi-square value	p-value
	Good n (%)	Average n (%)	Poor n (%)		
Faculty	5 (9.8)	32 (62.7)	14 (27.5)	18.5	0.005
Non-academic service/practice	1 (1.9)	24 (45.3)	28 (52.8)		
SR/JR/PG/Intern	0 (0.0)	37 (56.9)	28 43.1)		
Medical student	0 (0.0)	29 (54.7)	24 (45.3)		

A multiple linear regression analysis was also conducted to assess the associations of settlement status and source of food (independent variables) with HPS (dependent variable). The overall regression model was statistically significant (F-test, p < 0.001) (R² = 0.137), indicating that it explained 13.7% of the variance in HPS. Table 6 shows that settlement status was not a significant determinant of HPS (β = -0.071, p = 0.838), as there was no meaningful difference in HPS between trainees and settled individuals. In contrast, the source of food emerged as a significant predictor. Individuals consuming food from the hostel mess had significantly lower HPS compared to those consuming home-cooked food (β = -1.231, p < 0.001) (Table 6).

**Table 6:** Regression coefficients for predicting healthy practice scores Unstandardised β; Reference categories: Settled = 0, Home Kitchen = 0.

Predictor	β (Estimate)	SE	t	p-value	95% CI
Intercept	6.697	0.364	18.415	< 0.001	(5.980, 7.414)
Settlement (Trainee vs. Settled)	-0.071	0.345	-0.205	0.838	(-0.750, 0.609)
Source (Hostel vs. Home Kitchen)	-1.230	0.345	-3.563	< 0.001	(-1.911, -0.550)

## Discussion

This community-based cross-sectional study confirms that adequate nutritional knowledge and appropriate practices are lacking even among medical professionals, especially young doctors. Despite their medical background, the majority of participants lacked comprehensive nutritional knowledge, achieving only average scores. Awareness related to changes in guidelines was comparatively stronger, with nearly 7 of 10 participants being familiar with current NIN recommendations, indicating an overemphasis on updates instead of holistic education. Female participants, senior professionals, and older respondents consistently outperformed their peers across knowledge and awareness measures. Healthy dietary practices, however, remained inadequate among the participants. As expected, healthy eating practices were influenced by the source of food, with participants consuming home-cooked meals demonstrating better adherence to NIN recommendations.

We ensured balanced representation from both trainees and practising professionals while also capturing dietary sources (home-based and mess-based), which are often overlooked. Most existing studies [14-16] explored nutritional knowledge either among medical students or working professionals; to the best of our knowledge, they rarely examined and compared both groups together. Exploring the dietary behaviour of medical students and practising professionals has the potential to identify and pinpoint critical points of intervention with confidence during their medical careers.

Our study showed comparable female and male participation, with women demonstrating greater nutritional knowledge than their male counterparts. A comparable distribution of both genders represents the changing landscape of medical education in India, from male predominance to near-equal female participation in the medical curriculum in a previously male-dominated state, Uttar Pradesh, India. This shift has important implications for public health, as it provides a greater opportunity to impart nutritional knowledge effectively to both men and women, as nutritional decision-making is no longer confined to a single gender. Traditionally, higher nutritional knowledge across cultures, as confirmed in our study, when combined with adequate awareness rates among males, may have greater health benefits. Thus, the growing presence of both men and women in the medical fraternity offers a valuable opportunity to strengthen nutritional understanding.

Older participants and senior consultants (experienced in the medical field) demonstrated significantly higher NKS, consistent with findings among health care professionals in Saudi Arabia, Israel and Lebanon [19-21]. The inclusion of teaching faculty in the older age group, whose professional responsibilities necessitate staying up to date with current nutritional knowledge, is one reason. In contrast to findings from our study, low nutritional awareness was found among the elderly in the Korean population [22]. Nutritional knowledge should be established adequately from an early age, irrespective of medical or non-medical background, and sustained across the lifespan to maximise the health benefits, such as prevention of obesity, non-communicable diseases (NCDs), and cardiovascular events. This is crucial for reducing the overall burden of NCDs in India.

Superior knowledge does not always translate into better practice, as evidenced by our study findings and the published literature [14-16]. This discrepancy may be attributed to the presence of one or more factors that undermine the adoption of healthy behaviours. For instance, unmanaged academic stress may lead to meal skipping, followed by the consumption of UPFs due to their higher availability, reduced control over meal sources, and diminished emphasis on physical activity, including practices such as yoga [23,24]. Doctors occupy a pivotal role in shaping society’s health landscape, and hence should not only have sound nutritional knowledge but also practice it diligently to ensure effective adoption of lifestyle modification measures.

The source of food is also vital. We observed that students and older practising clinicians who regularly consumed home-prepared meals exhibited markedly healthier dietary practices compared to their counterparts. This observation underscores the role of supportive environments, wherein access to healthy food - reinforced by intrinsic motivation or structured guidance (either from home or self) results in superior dietary adherence [23,25]. The intergenerational transmission of food knowledge, as highlighted by Mengi Çelik et al., further suggests that home food environments serve as a foundational determinant of long-term nutritional behaviour [4,25,26].

Conversely, the importance of ensuring the quality of food from sources such as institutional canteens is often overlooked. These facilities function as a primary source of daily nutrition for millions, who frequently suffer from both immediate (cognitive function and energy levels) and long-term effects (micronutrient deficiencies and compromised immune function). The worldwide importance of ensuring healthy food for schools and institutions has been emphasised, and recommends their rigorous implementation be ensured [27]. Although regulatory frameworks governing institutional food environments exist in India, their implementation remains inconsistent and inadequate. Furthermore, habits formed during hostel years often persist into adulthood, with implications not only for long-term health but also for academic performance [28]. Therefore, it's vital to ensure equitable nutritional access across both home-dependent and institutionally-reliant populations.

Nutritional awareness scores were more than average in our population, but interestingly, awareness of current changes in dietary guidelines was present in similar proportions across the population, irrespective of their age, gender, and seniority. This implies an unbalanced nutritional education program for medical professionals, where much emphasis is placed on updates rather than holistic education. These lacunae in the nutritional education program for medical students are highlighted by Macaninch et al. [8,29]. The need to integrate nutritional education into the curriculum and to continue professional development on nutrition has been stressed [14-17]. For Indian medical graduates, the National Medical Council (NMC) launched the Competency-Based Medical Education (CBME) framework in 2024, which, when rigorously implemented, will allow nutrition teaching to be woven across preclinical, clinical, and community postings, reinforced through early clinical exposure [30]. Such an approach fosters both retention and application of nutrition knowledge in real-world patient consultations.

Strengths and limitations

To the best of our knowledge, this is a unique study exploring nutritional knowledge, awareness of updated guidelines, and practices across multiple strata of medical professionals. However, our study has certain limitations. One major drawback is that the participants were drawn from a single medical college; thus, the results may not be generalised to the entire medical fraternity regarding their understanding and practice of the nutritional guidelines. However, we tried to incorporate different groups to improve the quality of the data collected. Further studies across diverse settings and regions of the country are needed to ensure better representation and broader applicability of the results. Additionally, fairly proportional representation was achieved across multiple strata, which may have resulted in bias in the findings; we tried to overcome this by increasing the sample size using the design effect.

## Conclusions

In conclusion, this study demonstrates that nutritional knowledge remains insufficient and is inconsistently translated into practice, particularly among trainees, highlighting a critical gap. Systemic deficiencies in medical education and institutional food environments contribute to misaligned nutritional behaviour in the medical fraternity. Addressing this gap necessitates a multipronged approach encompassing curriculum reform, structured clinical nutrition training, policy-level intervention, and the creation of supportive dietary environments within academic and healthcare institutions. Strengthening nutrition competency within medical education is therefore not merely an academic imperative but a foundational investment in the long-term health of both the profession and the populations it serves.

## Appendices

Appendix 1. Study questionnaire

This study questionnaire collects information to assess the knowledge, aptitude, food habits and lifestyle of medical professionals associated with GS Medical College. Please provide the information requested. You are free not to participate or withdraw from the study at any time. Please do not write your identification details anywhere. The information collected shall be used exclusively for the study purpose, which has been approved by the college Institutional Ethics Committee.

Please tell us about yourself

1. Age: (Completed Yrs) ______

2. Gender: □ M     □ F       □ Other

3. Your responsibilities:

□ Faculty                □ SR/JR/PG/Intern        □ UG Medical Student             □ Other

4. You take food mostly (4 times or more in a week) from:

□ Home Kitchen           □ Hostel mess

5. Does anyone in your family suffer from lifestyle disease (please tick in the appropriate box)

**Table 7:** Does anyone in your family suffer from lifestyle disease (please tick in the appropriate box)

Disease	Self	Parents	Sibling	Spouse
Hypertension				
Diabetes mellitus				
Ischemic heart disease				
Any other				

Nutritional Knowledge

1. Nutri Cereals and legumes are good sources of Essential Fatty Acids

□ Correct         □ Incorrect       □ Don’t know

2. Refined oils have better nutritional value than non-refined oils

□ Correct         □ Incorrect       □ Don’t know

3. Non-stick (Teflon-coated) cookware is good as it can withstand high temperatures

□ Correct         □ Incorrect       □ Don’t know

4. Oils containing n-6 PUFA (like safflower) are good and can be taken in plenty

□ Correct         □ Incorrect       □ Don’t know

5. Cut vegetables should be thoroughly washed before cooking

□ Correct         □ Incorrect       □ Don’t know

6. Fortifying ultra-processed foods can make them wholesome and healthy

□ Correct         □ Incorrect       □ Don’t know

7. Spices are essential for good health due to their nutritional value

□ Correct, they have nutritional value   □ Incorrect, they provide taste only

8. Sunflower oil is not a good source of n-3 PUFA

□ Correct         □ Incorrect       □ Don’t know

9. The size of the sprout for the best nutritional value should be equal in size to the kernel.

□ Correct         □ Incorrect       □ Don’t know

10. Tea / Coffee can be taken in moderation

□ Correct         □ Incorrect       □ Don’t know

11. Tea/coffee should be avoided for one hour after a meal

□ Correct         □ Incorrect       □ Don’t know

12. Dietary fibre is nutritionally inactive

□ Correct          □ Incorrect       □ Don’t know

13. Microgreens are good sources of Amino Acids & Fatty Acids

□ Correct         □ Incorrect       □ Don’t know

Please answer the following questions (14-22) keeping in view the ICMR-NIN 2024 Guidelines

14. Table sugar is an essential dietary component

□ Correct         □ Incorrect       □ Don’t know

15. Commercially available ‘Mixed fruit juices’ have been recommended

□ Correct         □ Incorrect       □ Don’t know

16. Ready-to-eat bakery products are a good source of nutrition and are recommended

□ Correct         □ Incorrect       □ Don’t know

17. Baking soda can be added to pulses to facilitate proper cooking

□ Correct         □ Incorrect       □ Don’t know

18. In the food pyramid, Fruits & Vegetables form the base

□ Correct         □ Incorrect       □ Don’t know

19. A healthy adult is recommended to consume 400 gm of vegetables per day

□ Correct         □ Incorrect       □ Don’t know

20. A healthy diet and physical activity can reduce the risk of developing diabetes by 80%

□ Correct         □ Incorrect       □ Don’t know

21. Earthenware cookware has been considered the best.

□ Correct         □ Incorrect       □ Don’t know

22. Indians consume fewer vegetables than recommended

□ Correct         □ Incorrect       □ Don’t know

Preferences & Practice

1. What do you prefer?

□ Fruit Juice     □ Fruit

2. How many times do you consume Bakery products / Halwai-made products in a week?

□ Less than once          □ 1-3 times      □ 3-6 times      □ Daily

3. How many times in a week do you go for a brisk walk / moderate exercise lasting more than 45 minutes in a week?

□ Less than once          □ 1-3 times      □ 3-6 times      □ Daily

4. How often do you do yoga?

□ Less than once          □ 1-3 times      □ 3-6 times      □ Daily

5. How many cups of coffee / Tea you consume each day?

□ Do not take   □ 1-2 cups        □ 3-4 cups        □ 5 or more

6. Do you regularly consume sprouted beans?

□ Never           □ occasionally  □ 1-3 times per week    □ More than 3 times per week

7. How many times do you order food from outside in a week?

□ Less than once          □ 1-3 times      □ 3-6 times      □ Daily

8. How many times do you go out to eat (besides ceremonial gatherings) in a week?

□ Less than once          □ 1-3 times      □ 3-6 times      □ Daily

9. Which cooking medium is mainly used in your kitchen? (More than one answer accepted)

□ Mustard        □ Soybean        □ Canola          □ Coconut

□ Vanaspati      □ Ghee            □ Palm             □ Rice bran

□ Sunflower     □ Groundnut    □ Peanut          □ Olive
